# Using general practitioners with an extended role in spinal practice for the initial assessment of patients referred to spinal surgeons: preliminary experience and challenges

**DOI:** 10.1017/S1463423622000494

**Published:** 2023-01-26

**Authors:** Ian R. Whittle, Derek Yull, Josh Yee, Gus Czechowicz, Peter Murphy, Eleanor Clausen, YH Yau

**Affiliations:** 1 The International Spine Centre, Norwood, South Australia, Australia; 2 Faculty of Health and Medical Sciences, The University of Adelaide, Adelaide, South Australia, Australia; 3 Centre for Clinical Brain Sciences, The University of Edinburgh, Edinburgh, Scotland, UK

**Keywords:** general practice, low back pain, neck pain, spinal surgery, triage

## Abstract

**Aim:**

To describe experience using general practitioners (GPs), with an extended role (GPwER) in spinal medicine, to expedite assessment, triage, and management of patients referred from primary care for specialist spinal surgical opinion.

**Background:**

Low back and neck pain are common conditions in primary care. Indiscriminate or inappropriate referral to a spinal surgeon contributes to long waiting times. Previous attempts at triaging patients who really require a surgical opinion have used practice nurses, physiotherapists, clinical algorithms, and interdisciplinary screening clinics.

**Methods:**

Within the setting of an independent spinal care centre, we have used GPs specially trained in spinal practice to expedite the assessment and triage of new referrals between 2015 and 2021. We reviewed feedback from a Patient Satisfaction Questionnaire and the postgraduate backgrounds, training, practice with regard to triage of new referrals, and experiences of the GPs who were recruited

**Findings:**

Six GPwER had a mean of 26 years of postgraduate experience before appointment (range 10–44 years). The first four GPwER, appointed between 2015 and 2018, underwent an ad hoc in-house, interdisciplinary training programme and saw 2994 new patients between 2016 and 2020. After GPwER, assessment in only 18.9% (range 12.6 to 22.7%) of these patients was a spinal surgical opinion deemed necessary. Waiting times to see the spinal surgeon remained at 6–8 weeks despite a three-fold annual increase (from 340 to 1058) in new referrals. A Patient Satisfaction Questionnaire revealed high levels of satisfaction with the performances of the GPwER across seven dimensions. A dedicated training programme was designed in 2020, and the last two appointees underwent 20 h of clinical teaching prior to practice. Initial experience using GPwER, here termed ‘Spinal Clinicians’, suggests they are efficient at screening for patients needing spinal surgical referral. Establishing a recognised training programme, assessment, and certification for these practitioners are the next challenges.

## Introduction

Patients with low back pain (LBP) and neck pain (NP) constitute one of the top 10 cohorts of clinical presentations to Australian general practitioners (GPs) (Cooke *et al*., 2013; Bardin *et al*., 2017). Although there are evidence-based Guidelines and Protocols outlining management strategies for these patients in primary care (O’Connell *et al.*, 2016; Bardin *et al*., 2017; Traeger *et al.*, 2017; Oliveira *et al.*, 2018), many GPs are uncertain about current best practice (Buchbinder *et al*., 2009). This may be because of difficulties with Continuing Professional Development (CPD) in this area or competing demands in other areas of practice. Nonetheless, this uncertainty can lead to divergence from guidelines with the overuse of spinal imaging (Buchbinder *et al*., 2009; Traeger *et al.*, 2017; Wheeler *et al.*, 2018; Downie *et al*., 2020), injudicious and inappropriate prescription of analgesia (Buchbinder *et al.*, 2009; Traeger *et al*., 2017; Mathieson *et al.*, 2018; Foster *et al.*, 2018), inappropriate advice about rest and exercise, and unnecessary referral to a spinal surgical specialist (Buchbinder *et al.*, 2009; Foster *et al*., 2018; Buchbinder *et al.*, 2018). The latter problem adds to extended delays in patients who genuinely require more urgent spinal surgical consultation (Zarrabian *et al.*, 2017).

Although LBP and NP are common (Buchbinder *et al.*, 2018; Foster *et al*., 2018; Oliveira *et al*., 2018), undergraduate medical education about their pathophysiology, investigation, and treatment focuses on the 1% of disorders that have an underlying significant spinal pathology or the 5%–10% with myeloradicular syndromes (Bardin *et al.*, 2017; Traeger *et al.*, 2017). The ‘red flag’ symptoms and signs associated with these conditions are well taught, but paradoxically are often of dubious validity (Verhagen *et al*., 2016; Traeger *et al.*, 2017). Furthermore, clinical demonstration of many clinical signs requires a high level of clinical competence. Many signs are subtle, and although meaningful to a Neurologist, Neurosurgeon or Spinal surgeon can legitimately be missed by a GP. However, even in specialist practice errors can occur. In one series of proposed spinal operations, 11% were contra-indicated since the cause of the problem was not spinal (Lenza *et al*., 2017).

The problems of LBP/NP assessment in primary care and inappropriate secondary referral have been addressed by attempting greater education for primary care GPs about spinal medicine and advocating many different triaging and screening systems (Klein *et al*., 2000; Schectman *et al.*, 2003; Fourney *et al.*, 2011; Kindrachuk and Fournery, 2014; Lin *et al*., 2016; Zarrabian *et al*., 2017; Master and Hogarth, 2019; McKeag *et al*., 2020). Seven years ago, in an innovative attempt to provide more rapid access and triage of patients referred to a spinal surgeon from primary practice, we developed a novel intermediate grade medical practitioner. This practitioner is a GP with an extended role (GPwER) in spinal medicine (Gervas *et al.*, 2007). We have termed this practitioner a Spinal Clinician (SClin). Here we describe six GPs who have devoted several sessions of clinical practice a week to spinal medicine. They have done this within a private practice centre that focuses on the interdisciplinary management of patients with spinal disorders. The objectives of this paper are to describe the development of their clinical roles, practice, and educational needs; the relevance and advantages of this model; and their challenges for postgraduate education, assessment, and certification.

## Methods

### Study design

Description of a novel practice role and **r**etrospective cohort survey

### Setting

Private Single Site, inner suburban Australian capital city, Interdisciplinary Spinal Centre

The backgrounds of six GPs who have worked at the private spinal centre, on a sessional basis as ‘Spinal Clinicians’ (SClins), are described. The development of their clinical roles in the assessment and triage of new patients, their patterns of practice, mentorship, and CPD to prepare them for this role are outlined. Some benefits and disadvantages of having, and being, a GP SClin are highlighted. The challenges of validating and extending this role into primary care are discussed. Only the six GPs that have worked at the spine centre and no other doctors or practice have been analysed as part of this research.

Feedback from a Patient Satisfaction Questionnaire, based upon the Short Assessment of Patient Satisfaction (SAPS) measure (Hawthorne *et al*., 2014), that consisted of seven questions covering the performance of the SClins was audited to assess patient satisfaction with the SClins. Scoring of the each of the questions was based on a five-point Likert scale (0–4) with overall levels of satisfaction derived from the cumulative score from the seven items, graded according to a supplementary report from Hawthorne and colleagues (The SAPS) (continence.org.au)

## Results

The role of the SClins was the assessment, triage, and management of new patients referred to a Spinal Surgeon from either primary care or Insurance Companies dealing with work or vehicle injured employees. Specific goals of assessment were to identify those patients who had signs, symptoms, or imaging suggesting that referral to the Spinal surgeon would be of benefit. Most patients (90%:95% confidence limits 83%–95%, Tennant I, unpublished data) had undergone some form of spinal imaging in primary care.

Between Oct 2015 and December 2021, six GPs (mean time since graduation 26 years; range 10–44 years) opted to take up clinical sessions at a private the spinal centre. Their background qualifications, postgraduate experience, and number of sessions devoted to spinal medicine are listed in Table 1. Their sequential recruitment was consequent to the steady increase in referrals to the centre. Between Jan 2016 and December 2019, the first 4 SClins saw 2994 new patients (340 in 2016 increasing to 1058 in 2019). Due to the disruptions to clinical services caused by the COVID pandemic, data in 2020 and 2021 were not analysed. Three of these SClins had almost identical practice in terms of assessing the percentage of patients needed to be seen by the spinal surgeon (range 19.7% to 22.7% of new patients), whereas one referred significantly (*P* < 0.012) fewer (Table 1). The average waiting time to be seen was between 1 and 2 weeks, whilst during the same period the average waiting time to see the spinal surgeon varied between 6 and 8 weeks.

**Table 1.** Details concerning year of the general practitioners’ primary medical qualification, when they started at the Spine Centre, number of sessions devoted to spinal practice and the percentages of patients seen that were subsequently referred to a spinal surgeon. Five of the Spinal Clinicians were fellows of the Royal Australasian College of General Practitioners and two members of the Royal College of General Practitioners UK

Spinal clinician	Primary qualification	Sessions per week	When started as GPwER	Previous subspecialty interests	% of patients referred to Spinal Surgeon
01	MBChB 1982	3.25*	Oct 2015	Sports medicine	22.7
02	MBBS 1992	6.0**	July 2016	Functional assessments/work assessments	19.7
03	MBBS 1973	2.25*	Oct 2017	Disability medicine	20.7
04	MBBS 2008	5.5^*^	Aug 2018	Orthopaedics	12.6#
05	MBBS 1994	4.0^*^	May 2021	Pain Medicine	N/A
06	MBBS 1999	2	Feb 2022	Intensive Care	N/A

Abbreviation: GPwER general practitioner with an extended role.

* Indicates 2 or **8 spinal surgery theatre sessions/month.

# 04 referred significantly fewer patients to the spinal surgeon compared to the others (*P* < 0.012).

For the circa 80% of patients not initially referred by the SClins for surgical opinion, a management plan was formulated, often in collaboration with other members of the interdisciplinary team (Spinal Surgeon, Exercise Physiologist, Physiotherapist, Dietitian, Psychologist, Pain Specialist, General Physician, Rehabilitation Physician) within the centre. These patients were then either referred back to their own GP or followed up in house. Those patients who lived outside the metropolitan area had a management plan designed to utilise local facilities and were reviewed on an *ad hoc* basis. Patient Satisfaction Questionnaire responses from 51 patients revealed the mean score out of a possible 28 was 25.9 (standard deviation 2.63; median 27 and 95% confidence interval 25.2 to 26.6). All patients were either satisfied (*n* = 20, score between 19 and 26) or very satisfied (*n* = 31, score 27–28) with their consultation (Table 2).

**Table 2.** Answers to the seven questions concerning the performance of the Spinal Clinicians that were included in a patient satisfaction questionnaire. Responses were obtained from 51 new patients seen either in 2021 or 2022. Patients were asked either to give their feedback via an APP or on a dedicated tablet following their Consultation

0	1	2	3	4
Strongly	Disagree	Neutral	Agree	Strongly
Disagree		Neither agree/disagree		Agree
Q1: Overall I was satisfied with the outcome of my Consultation				
0	0	0	14 (27%)	37 (73%)
Q2: Compared to my normal GP I feel the SClin exhibited increased expert knowledge				
0	0	0	13 (25%)	38 (75%)
Q3: I was satisfied with the options discussed in my treatment pathway				
0	0	0	15(29%)	36 (71%)
Q4: The time I spent with the Dr/SClin was about right				
0	0	0	20 (39%)	31 (61%)
Q5: The SClin showed genuine interest in my problems				
0	0	0	12 (24%)	39 (76%)
Q6: I was able to express my problems/concerns				
0	0	0	15 (29%)	36 (71%)
Q7: The Dr/SClin made me feel welcome and put me at ease when I first met				
0	0	0	15 (29%)	36 (71%)

Patient satisfaction questionnaire feedback.

All questions were based on a 5-point Likert scale.

Since the appointment of a Director of Research and Education at the Centre in Jan 2020, a formal curriculum encompassing Basic Clinical Sciences related to the spine, Spinocentric History Taking and clinical examination, Basic Spinal Pathophysiology and Pathology, Spinal Imaging, and Management Strategies has been established with dedicated assessments of the GP’s knowledge after each module. The last two SClins appointed therefore underwent a total direct contact teaching time of > 20 h each. This has complemented the pre-existing in-house multi-dimensional professional educational activities already available. CPD is also facilitated by formal monthly evening in-house training sessions lead by the Spinal Surgeon that *inter alia* involved case reviews, interpreting spinal imaging, reviews of journal articles, and feedback about aspects of the job. There were also bimonthly interdisciplinary team meetings that consolidated personal learning about spinal care. Patient feedback about the competence and skills (of the SClins compared to their GPs) revealed that all patients either agreed (26%) or strongly agreed (74%) that the SClins had greater knowledge of spinal medicine (Table 2). All six SClins have continued to practice some sessions as GPs in Primary Care medicine out with their Spine Centre roles.

## Discussion

The role of a GPwER in spinal medicine within a dedicated private practice centre seems a novel concept (Yellamaty *et al.*, 2019). Its planned development followed the increasing patient volume referred to the centre that was leading to prolonged waiting times to see the Spinal Surgeon. Many of these patients with LBP and NP referred from primary care do not need referral to a spinal surgeon, since most of these patients (circa 90%) do not have a surgical cause for their pain (Fourney *et al.*, 2011; Bardin *et al*., 2017; Foster *et al*., 2018; McKeag *et al.*, 2020). They do however require assessment and a management plan based upon evidence-based guidelines (Traeger *et al*., 2017; Foster *et al.*, 2018; Oliveira *et al*., 2018; Mckeag *et al*., 2020). A desire to establish interdisciplinary care pathways for this large cohort was an additional consideration given that advice about spinal conditions given by some GPs is suboptimal (Oliveira *et al*., 2018).

All the SClins found their clinical sessions stimulating and rewarding. The amount of time available to assess a new patient (30–45mins), compared with routine General Practice, was considered a significant benefit. Appointment times in General Practice are usually limited to 10 to 15 min for a standard consultation. The length of the consultations was universally appreciated by patients (Table 2). The SClins also had dedicated theatre sessions at which they assisted the Spinal Surgeon during surgical procedures on patients they had assessed. This opportunity was enjoyed since it enabled correlation of clinical MRI and operative findings.

The SClins contrasted their practice at the Spinal Centre setting for LBP/NP patients with the isolation of being a GP, where time constraints, potential litigation, and workers compensation patients were deemed problematic. Another benefit of having GP SClins triaging new patients was that their diverse interests and diagnostic skills enabled early recognition of non-spinal conditions (eg, hip problems) and a range of esoteric diagnoses to be made. These included autoimmune disorders, demyelinating conditions, metastatic cancer, osteitis pubis, and two cases of the Foix-Alajouanine syndrome.

The concept of subspecialisation for GPs is not new, and these practitioners were initially termed GPs with a special interest and subsequently GPs with an extended role (Gervas *et al.*, 2007; Taneja *et al.*, 2015; Yellamaty *et al.*, 2019). Principal issues that these appellations raise in many areas of practice relate to quality of service provided, impact on waiting times, outcomes, patient satisfaction, costs, and attitudes developed (Gervas *et al*., 2007; Taneja *et al.*, 2015; Yellamaty *et al.*, 2019). Supportive data for GPwER may be specific, such as the audit of Australian GPs specialising in the use of Dermatoscopes for the diagnosis of malignant melanoma that showed they halved the number of patients needed to be treated for each correct diagnosis (Rosendahl *et al.*, 2012) or, as in this paper, from generalised results of audit (Buchbinder *et al.*, 2009). In our series, other supportive data were more empiric. The waiting time to see the Spinal Surgeon was constant despite a three-fold increase in patient referrals. Additionally, the percentage of new patients that were subsequently referred by each SClin to the spinal surgeon (range 13%–23%) is in keeping with the range of 10% and 28% of new LBP patients referred by specially targeted GPs and trained physiotherapists (Kindrachuk and Fourney, 2014; McKeag *et al.*, 2020) and the widely accepted figure of between 80% and 90% patients that could be managed non-operatively (Bardin *et al.*, 2017; Traeger *et al*., 2017; Foster *et al*., 2018).

The feedback from the patient satisfaction Questionnaires is also supportive of the professionalism and skills of the SClins. The Questionnaire used in this study was a modification of the SAPS, a measure that has very good reliability (Cronbach’s alpha 0.86) and psychometric properties (Hawthorne *et al.*, 2014). Five of the seven questions asked were either identical or very similar to the SAPS whilst one required changing (effect of surgical treatment was not appropriate to this study), and the technical skills aspect of the SAPS was altered to reflect differences in skills between the SClins and GPs. The modified Questionnaire covered all essential dimensions stated by Hawthorne (Effectiveness, Information, skills, participation, relationship, access and facilities, and general satisfaction). Generally, between 70% and 90% of patients are satisfied with medical consultations, but patient satisfaction is a ‘notoriously slippery concept’ (Hawthorne *et al*., 2014) with many factors influencing it (Grogan *et al.*, 2000; Dwamena *et al*., 2012).

It may seem a paradox to have GPs at a spinal centre assessing and triaging patients referred by GPs in primary care. One could argue that this unnecessarily complicates management and referral patterns and causes additional delays and costs (Yellamaty *et al.*, 2019) as well as frustrating the referring GPs. However, many referring GPs were confident that their patients did not need spinal surgery, but they were unsure of optimal management (Buchbinder *et al.*, 2009; Bardin *et al*., 2017; Traeger *et al.*, 2017) and thus, through the portal of the SClins, sought guidance and reassurance from the interdisciplinary team at the spinal centre. There is also evidence that focussed education of GPs in guidelines about LBP decreases both referral rates to spinal surgeons and incidence of radiological imaging (Schectman *et al.*, 2003; Kindrachuk and Fourney, 2014; Lin *et al*., 2016). The SClins were also happy, unlike many GPs, to accept Workers Compensation cases (Brijnath *et al.,* 2016) because of the interdisciplinary support facilities readily available. They are also well placed, compared to physiotherapists performing a primary assessment and triage role (Fourney *et al.,* 2011), to consider both wider medical diagnoses and the psychological component of LBP/NP due to their postgraduate GP medical and mental health training. In all cases, they liaised directly with the referring GP or corporate body with correspondence outlining their findings, opinions, and management strategy.

This raises the question as to what defines a GP SClin because currently there is no recognised certificate or diploma of competency in this field from a regulatory body. This is a problem with GPwER in many disciplines (Yellamaty *et al.*, 2019). This novel appellation was bestowed upon the four GPs during 2020 when it became apparent that through their commitment to the role, their progressive assimilation of knowledge through multifaceted learning, and competency in clinical skills related to spinal medicine, they had become a uniquely skilled GP. This assessment was confirmed by the feedback from the PSQ and would also seem justified from previous studies outlining shorter and more focussed periods of GP education that had positive outcomes on managements strategies and adherence to guidelines (Klein *et al.*, 2000; Schectman *et al.*, 2003; Lin *et al*., 2016).

Although the six GPs appointed did not have specific training in spinal medicine prior to practising as a SClin, they were all very experienced GPs and had background training, experience, and interests in orthopaedics, sports and musculoskeletal medicine, pain medicine, and disability assessment (see Table 1). These factors no doubt facilitated their transition into the SClin role where in-house training consolidated their knowledge and skills. Ideally, these skills and knowledge should be attained within a structured setting since previous experience of GPs self-proclaiming specialist knowledge in spinal medicine has not withstood scrutiny (Oliveira *et al.*, 2018). With this in mind, a dedicated curriculum and teaching programme was developed so that the latter two appointees were well prepared for their new practice roles. All six doctors felt their knowledge of spine medicine increased dramatically, and this was not at the expense of their generalist approach to medicine. It is recognised that LBP is a condition and not a disease, may be recurrent and is intimately linked to psycho-socio-economic factors. Feedback from GP colleagues suggests they value working with doctors who have a special interest in spine medicine as this can provide educational opportunities as well as assistance with managing their patients.

Ideally to establish a firm foundation for the role of a GPwER in Spinal Medicine, both CPD requirements of knowledge, skills and attitudes, and their assessment and certification by a reputable regulatory body are required. The Royal College of GPs (UK) had defined what competencies and skills were required for 17 different subspecialties to be registered or qualified as a GPwER. However, since the dissolution of Primary Care Trusts in 2013, there are currently only guidelines for the subspecialty of dermatology (https://www.rcgp.org.uk/training-exams/practice/general-practitioners-with-extended-roles.aspx; last accessed 10/10/21). The Royal College of GPs (UK) also state that they do not have the ‘operational capacity to provide GPwER assessment’. The Royal Australian College of GPs does not mention GPwER on its website but does describe a certification course for GPs with a special interest in Dermatology in its Education section (https://www.racgp.org.au; last accessed 10/10/21).

The limitations of this descriptive study fall into several categories. The lack of outcome data for the 80% of patients managed by the SClins is problematic. Realistically, either qualitative or quantitative data would be difficult to obtain from an audit because of the heterogeneity and complexity of both this sample population and the patient-reported outcome measures that would be required. In one retrospective follow-up of patient outcome from an Australian interdisciplinary spinal clinic, only 39.9% of 88 patients gave feedback on their outcomes (Masters and Hogarth, 2019). Many of these patients also have chronic conditions so that ‘cure’ is unlikely. Additionally, because the majority of these patients are privately insured, they can readily seek alternative management opinions from other primary care physicians and health workers. Obtaining this follow-up information would be extremely difficult. We have also not addressed the related question of accuracy of diagnoses by the spinal clinicians. Again, this is difficult to address because of the patient cohort heterogeneity and because many of these patients defy definitive diagnosis and are labelled as having chronic LBP or chronic NP. Given the percentage of patients subsequently referred to the spinal surgeon (circa 20%), the facility for follow-up review at the spinal centre and the fact that up to 11% of operations planned by spinal surgeons may be erroneous (Lenza *et al.*, 2017) we feel misdiagnosis of significant spinal disorders would be small.

Another practical problem that has not been addressed is the applicability of this model to other settings such as primary care or the public health sector. To prevent the long waiting times seen in public sector specialist spinal clinics (Zarrabian *et al.*, 2017; Masters and Hogarth, 2019), SClins could be embedded in large Primary Care Centres or even to screen patients in Hospital outpatient clinics. Whether this would be deemed cost-effective would require an answer to the questions: (i) if I see a SClin will I still get the right treatment in the end; and (ii) will it be quicker and cheaper? Appointments to such positions would depend upon and require some training in the sphere of spinal medicine. However as discussed, no formal training programme exists. Gaining recognition for such a programme and its formal assessment requires a recognised Academic Medical body to become a stakeholder. This recognition would subsequently need to satisfy various employing institutions as well as other Academic Medical bodies that have a vested interests in aspects of SClin practice encroaching their deemed jurisdictions.
